# Immunological suppression of head and neck carcinoma by dendritic cell tumor fusion vaccine

**DOI:** 10.3892/ol.2013.1633

**Published:** 2013-10-15

**Authors:** YONGBIN MOU, HAO XIE, XIAOFENG HUANG, WEI HAN, YANHONG NI, HANG SU, ZHIYONG WANG, QINGANG HU

**Affiliations:** 1 Center Laboratory of Stomatology, Stomatological Hospital Affiliated Medical School, Nanjing University, Nanjing, Jiangsu 210008, P.R. China; 2 Institute of Life Sciences, The Key Laboratory of Developmental Genes and Human Disease, Southeast University, Nanjing, Jiangsu 210096, P.R. China

**Keywords:** head and neck carcinoma, dendritic cell, cell fusion, cancer vaccine

## Abstract

The successful treatment of cancer with dendritic cell (DC) tumor vaccine is highly dependent on the efficacy of antigen presentation and T cell activation. In the present study, a novel vaccine of DCs fused with autologous tumor cells was introduced, which had a marked ability to suppress head and neck carcinoma. DCs generated from the bone marrow of mice were fused with an autologous tumor cell line using polyethylene glycol (PEG). To observe the fused cells, confocal microscopy and FACS analysis were performed. Subsequently, the activation and proliferation of T cells, as well as animal experiments, were examined. The efficiency of DC/tumor fusion was 18.03% and T cells were well-activated by the hybrids. The volumes of tumors on the tumor-bearing mice were controlled, survival time of tumor-bearing mice was prolonged and the level of IFN-γ in serum was significantly increased compared with the control group and lysate-pulsed DC group. The results indicate that the DC/tumor fusion vaccine appears to be more effective than DCs pulsed with tumor lysate for the treatment of head and neck carcinoma, which may be useful in future clinical studies.

## Introduction

Tumor antigens expressed on the surface of malignant tumor cells serve as target proteins for the host immune system, which remains a major area of cancer research. Cancer vaccines of various types have highlighted possible approaches for treatments that enhance the patient’s own immunity. Dendritic cells (DCs) are particularly suitable for cancer vaccines, as they are potent antigen-presenting cells. DC-based antitumor vaccines have emerged as promising cancer immunotherapies with confirmed clinical efficacy (1,2).

Major therapies to achieve the goals for the cellular immunotherapy of cancer are divided into the following two groups: i) vaccination of the tumor-bearing host with tumor antigens to elicit T cell-mediated immunity to eradicate the established tumor; and ii) treatment of the malignancy via the adoptive transfer of *in vitro* cultured tumor-reactive T cells (3–5). However, the majority of malignant tumor cells are poorly immunogenic and are capable of evading the host immune surveillance. Previous results have demonstrated that tumor cells downregulate the expression of signals that are essential for the activation of host T cells. The mechanisms include defective expression of major histocompatibility complex (MHC) molecules, absence of co-stimulatory or adhesion molecules and alteration of antigen-processing or transport, which result in an inability to present tumor-associated antigens (6). Strategies of augmenting the host immune responses to the tumor included the introduction of genes encoding MHC molecules (7), co-stimulatory molecules (8) and cytokines (9) into tumor cells or DCs to improve immunogenicity and antigen-presenting capabilities.

DCs are uniquely capable of inducing primary immune responses (10). Accumulating data have shown that DCs induce marked antitumor immune responses *in vitro* and *in vivo* by their exceptional capabilities to capture antigens, process and present antigenic peptide fragments, migrate to lymphoid organs and strengthen the primary immune responses of CD8^+^ and CD4^+^ T cells (11).

As few human tumor antigens have been identified, immunization with DCs pulsed by purified tumor-associated peptides or proteins has been regarded as a limited strategy for clinical application. Due to a lack of tumor antigens, several strategies of using tumor cell-charged DCs as vaccines for cancer immunotherapy have been developed (5,12,13). It has been hypothesized that the fusion vaccine may have capabilities of antigen expression and presentation, and elicit effective antitumor immunity (14). In the present study, the fusion cellular vaccine of DCs and syngeneic tumor cells was investigated to identify whether it effectively elicits host antitumor immunity.

## Materials and methods

### Mice

Female C3H/HeJ mice were purchased from the Model Animal Research Center of Nanjing University (Nanjing, China) and fed under specific pathogen-free conditions in the Central Animal Facility, Nanjing University. In a typical experiment, bone marrow was isolated from the femur of 8- to 10-week-old mice, weighing 20–22 g. All animal experiments were performed in accordance with protocols approved by the Animal Care and Use Committee of the Medical School, Nanjing University.

### DCs culture and tumor cell line

DCs were generated according to methods described in our previous study (15). Briefly, monocytes were isolated from the bone marrow of mice. Marrow monocytes were flushed out from the femur and tibia, cultured with RPMI 1640 supplemented with 10% FBS (both Gibco-BRL, Carlsbad, CA, USA), 50 mM 2-Mercaptoethanol, 100 mM sodium pyruvate, 100 U/ml penicillin, 100 mg/ml streptomycin, 10 ng/ml recombinant GM-CSF and 1 ng/ml murine recombinant IL-4 (all eBioscience, Inc., San Diego, CA, USA). On days 2 and 4, 50% of the medium was removed and fresh media was added. The released non-adherent immature DCs (imDCs) were collected on day 6. SCC7, a mouse head and neck carcinoma cell line, was cultured in DMEM media supplemented with 10% FBS, 2 mM L-glutamine, 100 U/ml penicillin and 100 mg/ml streptomycin.

### DC/tumor fusion protocol

Bone marrow-derived DCs were fused with tumor cells at a DC to tumor ratio of 3:1 using 50% polyethylene glycol (PEG; molecular weight, 1450)/dimethyl sulfoxide (both Sigma-Aldrich, St. Louis, MO, USA) solution. Briefly, the DC/tumor cell suspension was washed twice with RPMI-1640 and prewarmed to 37°C. PEG (50%; 1 ml) was added over 1 min and the suspension was stirred gently for 1–2 min. Prewarmed RPMI-1640 (1 ml) was then added over 1 min and the suspension was stirred. An additional 3 ml-RPMI-1640 was added over 3 min, followed by 10 ml RPMI-1640, which was added slowly. Following incubation for 5 min at 37°C, the resultant cell mixture was pelleted and grown overnight in CM with GM-CSF. The fused cells were irradiated (50 Gy), prior to injecting into mice to render them non-proliferative.

### Confocal microscopy

In total, ~2×10^4^ DC/tumor fused cells were spread on the coverslip, and DC and tumor cells were stained with Cy5.5 and CFSE (Molecular Probes, Eugene, OR, USA), respectively. Cells were washed, fixed and analyzed using a Laser Scanning Confocal microscope (Fluoview, Fv10i; Olympus Corp., Tokyo, Japan).

### FACS analysis of DC/tumor cells

The post-fusion ratio of DC, SCC7 and DC/SCC7 fused cells was determined by FACS analysis. Briefly, 3×10^6^ DCs and 1×10^6^ SCC7 cells were incubated with Cy5.5 (10 μg/ml) and CFSE (5 μM) for 8 h, respectively and then washed with FACS buffer. The fused cells were collected and fixed with 1.0% paraformaldehyde. FACS analysis was performed on a FACS flow cytometer (BD Biosciences, Franklin Lakes, NJ, USA).

### Tumor lysate-pulsed DC preparation

DCs were pulsed with freeze-thawed tumor lysates at a DC to tumor ratio of 1:3. Briefly, SCC7 tumor cells (1×10^7^ cells in 500 μl RPMI) were lysed by repetitive rapid freezing in liquid nitrogen and thawing in a 37°C bath, which was repeated three times. Cellular debris were centrifuged (300 × g for 3 min) and only the lysate supernatants were used to pulse DCs. Pulsed DCs were harvested and washed twice following incubation for 12 h and then injected into mice.

### Activation and proliferation of T cells by mixed lymphocyte reaction (MLR)

DC/tumor fusion cells and DCs pulsed with tumor lysate were collected and washed. Purified CD4^+^ T cells were obtained by magnetic bead (Miltenyi Biotech, Bergisch Gladbach, Germany) method from the lymph nodes (axillar, inguinal and mesenteric) of normal C3H/He mice. Syngeneic T cells (1×10^5^/well) were incubated with fused cells or DCs pulsed with tumor lysate at the ratio of 10:1 in flat-bottomed 96-well plates. Following incubation for 3 days, the proliferation of T cells was determined by Cell Counting Kit-8 (Dojindo, Kunamoto, Japan) incorporation assay. By contrast, the same level of T cells were incubated with ConA (final concentration, 5 μg/ml).

### Animal experiments

For therapeutic experiments, mice received s.c. injection of 2×10^6^ SCC7 cells in the right flank on day 0. On day 7, mice were randomized into cohorts of eight mice, each exhibiting average tumor sizes of 20–30 mm^2^. On day 7, tumor-bearing mice were treated with s.c. injections in the two foot pads of 1×10^6^ DC/tumor cells or DCs pulsed with tumor lysate in a total volume of 40 μl PBS. On day 14, the two sides of inguinal lymph nodes were injected with the same number of cells as described previously. Tumor sizes were then assessed every 3 days, recorded in mm^2^ and determined as the product of orthogonal measurements collected using vernier calipers. The volume was calculated using the following formula: (short diameter)^2^ × (long diameter) × 0.52. Data are presented as the mean tumor area ± standard deviation (SD).

### Enzyme-linked immunosorbent assay (ELISA)

Following the sacrifice of animals, serum was separated. ELISA was performed using a mouse IFN-γ ELISA kit (Dakewe Biotech Co., Ltd., Shenzhen, China), according to the manufacturer’s instructions. Briefly, serums were added into the 96-well plates coated with IFN-γ monoclonal antibody in duplicate and incubated for 1 h at 37°C. Next, the plate was washed with washing buffer three times. The HRP-conjugated secondary antibody was then added into each well and the plate was incubated for 1 h at 37°C. After washing the plate three times, TMB substrate solution was added into each well and incubated for 15 min at room temperature in the dark. Subsequently, the stop solution was used to terminate the reaction. The absorbance was then determined at 450 nm by using a Synergy HT microplate reader (Bio-Tek Instruments, Inc., Winooski, VT, USA).

### Statistical analysis

Data are presented as the mean ± SD. Comparisons were conducted using one-way analysis of variance, and P<0.05 was considered to indicate a statistically significant difference.

## Results

### Confocal microscopy

**Figure 1:**
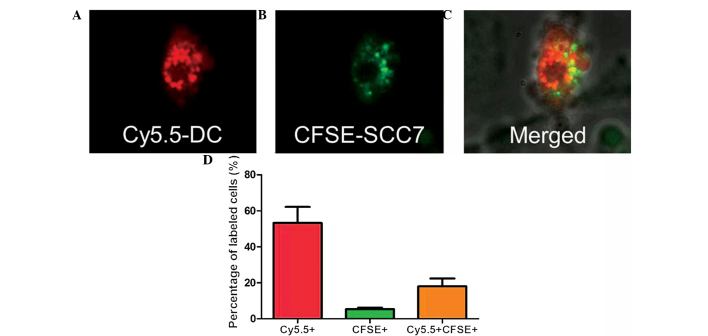
DCs labeled with Cy5.5 were fused with CFSE-SCC7 by PEG at a ratio of 3:1 and fusion preparations were cultured overnight. The representative fluorescence photomicrographs showed (A) DCs labeled with Cy5.5, (B) SCC7 cells stained with CFSE and (C) DC/SCC7 fused cells (magnification, ×2,400). (D) Fusion efficiency was determined by FACS and the results were counted. DCs, dendritic cells; PEG, 50% polyethylene glycol.

Fused cells were first characterized by confocal microscopy; DC surfaces were identified by Cy5.5 (red color; Fig. 1A), while tumor cells were identified as CFSE-positive (green cells; Fig. 1B). Overlapping these images resulted in the detection of a subset of cells with both colors (Fig. 1C), indicating DC/tumor fused cells. Following confirmation of the fusion of Cy5.5 and CFSE dual-labeled DC/SCC7 hybrids by confocal microscopy, cell suspensions were prepared to be measured by FACS and the percentage of DC/SCC7 hybrids was found to be 18.03%. Almost all tumor cells were involved in cell fusion, with ~4.99% unfused tumor cells remaining (Fig. 1D).

### Proliferation of T cells by MLR

**Figure 2:**
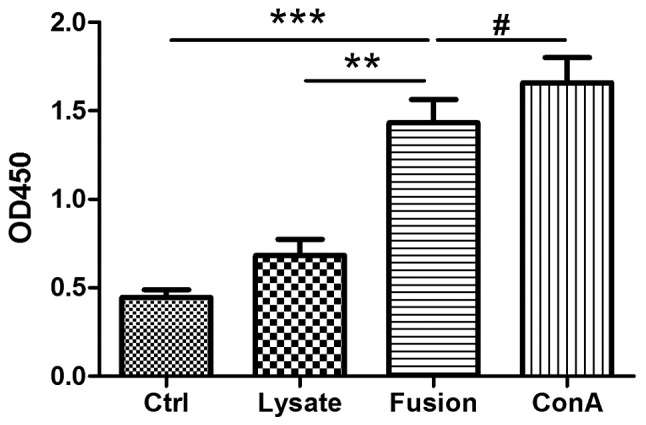
Mixed lymphocyte reaction of DC/SCC7 hybrids with syngeneic T cells. DCs derived from bone marrow and fused with SCC7 cells (ratio, 1:3) were co-cultured with syngeneic T cells for 3 days at a ratio of 1:10, and the proliferation of T cells was measured by the Cell Counting Kit-8 method. Absorbance was measured at an optical density of 450 nm. DCs, dendritic cells. ^#^P>0.05, ^**^P<0.001 and ^***^P<0.0001.

To confirm the capacities of antigen processing and presenting, naive syngeneic T cells were stimulated by DC/SCC7 hybrids and DCs loaded with SCC7 whole tumor lysate, which had been co-cultured with T cells at a ratio of 1:10 in a 3-day MLR. As shown in Fig. 2, DC/SCC7 hybrids and ConA evidently promoted T cell proliferation, whereas tumor lysate-pulsed DCs induced a smaller increase in proliferation following stimulation, compared with the controls. These results demonstrated that DCs and SCC7 hybrids have an improved ability to present antigens to T cells than the lysate group (Fig. 2).

### Antitumor efficacy of DC/SCC7 fused cells in SCC7 cancer model

**Figure 3:**
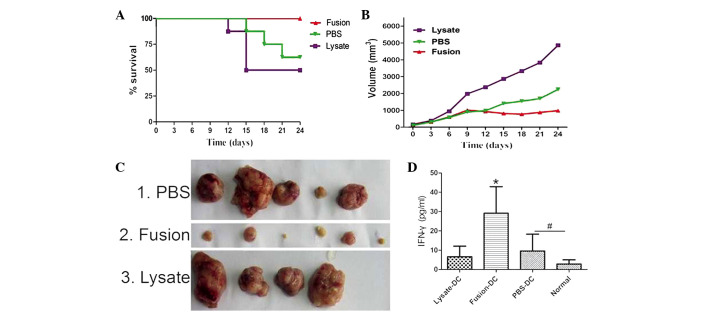
Immunization of C3H/HeJ mice with DC/SCC7 fusion vaccine elicits host resistance against tumor challenge. Mice were challenged by s.c. injection of 2×10^6^ SCC7 tumor cells. At days 7 and 14 following tumor implantation, tumor-bearing mice were vaccinated subcutaneously (s.c.) and intranodally (i.n.) with 2×10^6^ DC/SCC7 hybrids and DCs pulsed with lysate, respectively. (A) Survival time and (B) tumor growth in each group of mice were recorded. (C) Tumor size was determined by measuring the largest perpendicular diameter in mm^2^ with calipers and recorded every 3 days. (D) IFN-γ cytokine in the serum was determined by ELISA. ^*^P<0.05 and ^#^P>0.05. DC, dendritic cell; ELISA, enzyme-linked immunosorbent assay.

Mice bearing 7-day established SCC7 tumors were vaccinated (s.c.) with DC/SCC7 hybrids and DCs pulsed with lysate collected from parental SCC7 tumor cells. Mice treated with PBS served as controls. Vaccination with DC/SCC7 hybrids evidently improved survival rate (Fig. 3A) and markedly inhibited tumor growth (Fig. 3B), compared with that of DCs pulsed with lysate. In total, two out of eight treated mice were rendered tumor-free and all mice survived up to sacrifice (Fig. 3C). On the 24th day, mice were sacrificed and serum was collected and stored immediately at −20°C until analysis. The level of IFN-γ in the serum of the fusion group was higher compared with that of the other groups (Fig. 3D).

## Discussion

The immune response of DC vaccines against solid tumors has often been shown to be weak or localized and, therefore, there has been concern with regard to the requirement to develop a novel approach against tumors. The advantage of the DC/tumor fusion vaccine compared with pulsing DCs with tumor lysate may be that endogenously synthesized antigens have improved access to the MHC class I pathway (16). An additional important difference between the DC/tumor fusion strategy and the whole tumor lysates loading strategy, is that DCs, as well as tumor cells, are independently modified while their characters persist following fusion.

In the current study, bone marrow imDCs from 6-day culture in GM-CSF- plus IL-4-supplemented medium were used. It is known that imDCs have more active antigen processing capabilities compared with those of mature DCs (mDCs). However, whether tumor and imDC or mDC hybrids generate a more potent antitumor effect remains to be determined. In the present study, the TNF-α cytokine was not selected to stimulate the maturation of imDCs, which means the hybrids also have the ability to active T cells and initiate anti-tumor immune responses.

The results of the current study revealed that almost all tumor cells were fused with DCs and that only a few tumor cells, ≤5%, remained unfused. This implies that this type of fusion technology has a satisfied fusion efficiency. Efficient separation of the tumor/DC hybrids from parental tumor cells in the fusion preparations remains a technical challenge, therefore, the remaining unfused tumor cells were irradiated prior to being injected into mice. To confirm the capacities of antigen processing and presenting, naive syngeneic T cells were stimulated by the fusion vaccine and DCs loaded with tumor cell lysate, which had been tested by MLR assay. The results revealed that the hybrids significantly induced the proliferation of T cells.

In our previous studies, the migration of DCs labeled with super paramagnetic iron oxide particles and enhanced green fluorescent protein by magnetic resonance imaging and optical imaging demonstrated that DCs migrate into the cortical area of the draining popliteal lymph nodes by footpad injection (17). Notably, the ability of DCs to migrate to lymph nodes was limited following intravenous infusion (data not shown), and the antigen-specific immune responses induced by intranodal injection were similar to those of intradermal injection (18–20). Thus, footpad injection of the DC vaccine was selected as the primary immunity and intranodal infusion as the secondary dose one week later.

Previous animal studies have demonstrated that fusion vaccines are superior to DCs loaded with tumor cell lysates (21–23). Through cross-priming, the fusion cells activate antigen-specific CD4^+^ T cells that become multifunctional effectors, producing cytokines, particularly IFN-γ (24–26). Moreover, the fusion cells function as antigen-presenting cells with the ability to migrate to draining lymph nodes, where they reside in the T cell area, interact with CD4^+^ and CD8^+^ T cells and induce potent antitumor immunity (24,27).

In summary, the present study demonstrated that the novel cancer vaccine of DC/tumor hybrids promotes the proliferation of syngeneic T cells and elicits a clear antitumor T-cell response. The results indicate that the relatively crude preparations of DCs and tumor cells are likely to suffice as sources of DC and tumor fusion and, thus, indicate that DC/tumor hybrids may be of value for the treatment of OSCC. Meanwhile, the fusion conditions for DCs and tumor cells must be optimized for use in a clinical setting. The current study presents an investigation into the strategies to circumvent immune evasion of OSCC prior to the implementation of large-scale clinical assessment.
